# Regarding: Staples, tension-band plates, and percutaneous epiphysiodesis screws used for leg-length discrepancy treatment: a systematic review and proportional meta-analysis

**DOI:** 10.2340/17453674.2025.43005

**Published:** 2025-02-10

**Authors:** Bjoern VOGT, Adrien FROMMER, Georg GOSHEGER, Andrea Maria LAUFER, Robert RÖDL, Gregor TOPOROWSKI

**Affiliations:** 1 Pediatric Orthopedics, Deformity Reconstruction and Foot Surgery, Muenster University Hospital; 2 General Orthopedics and Tumor Orthopedics, Muenster University Hospital, Germany

*Sir,*—We are writing in response to the recently published review article, “Staples, tension-band plates, and percutaneous epiphysiodesis screws used for leg-length discrepancy treatment: a systematic review and proportional meta-analysis” by Tirta et al., in *Acta Orthopaedica*. This comprehensive review evaluates the success rates, effectiveness, and complications of various epiphysiodesis techniques in treating leg-length discrepancy (LLD) in the pediatric population [1].

While the review is a valuable contribution to the field, we would like to address some inaccuracies regarding our own work (Vogt et al., 2021, Reference 47), specifically concerning the RigidTack device, which we have developed [1]. First, it is methodologically incorrect to categorize the RigidTack with Blount staples. As detailed in our 2021 publication, the RigidTack system, consisting of a rigid staple with barbed and cannulated prongs for improved bone purchase and Kirschner-wire guided implantation, is fundamentally different compared with traditional Blount staples. Only 1 single device is needed per operation site and implant-associated complications such as loosening, migration, or breakage rates are significantly lower [2].

Additionally, the review’s calculation of the success rate for leg length correction using the RigidTack, as presented in Figure 4 and Tables 2 and 4, is incorrect [1]. Our original paper provides a much more nuanced analysis of success rates, which were misrepresented in the review [2]. This misrepresentation erroneously suggests that the RigidTack has the lowest success rate among all included implants, which is not the case.

Moreover, Tables 2 and 4 of the review incorrectly state that the initial and final LLD were not reported in our study [2]. In fact, these details are comprehensively documented in our paper. The initial LLD using the eight-plate was 26.7 mm and 11.0 mm after treatment (see “Results,” subsection “Comparison of rigid staples with two-hole plates and Blount staples”). The initial LLD using the RigidTack was 25.2 mm and 9.3 mm after treatment (see “Results,” subsection “Rigid staples”). In contrast, when using Blount staples, the initial LLD was 29.3 mm and 11.4 mm after treatment (see “Results,” subsection “Comparison of rigid staples with two-hole plates and Blount staples”). These results are also depicted in Figure 4 [2].

This oversight might lead readers to underestimate the thoroughness of our research and the effectiveness of the RigidTack system.

As already mentioned, please note that we have observed significant differences in complications between the RigidTack and the Blount staple [2], which is why these implants should not be grouped together in 1 category. The individual parameters can be found in the “Results” section, subsection “Complications” [2].

Despite these issues, we commend the authors for their significant effort in compiling this systematic review and meta-analysis. Their work provides crucial insights into the often uncritically applied treatment methods for LLD, highlighting both the overestimated success rates and underestimated complication rates of various techniques.

We would greatly appreciate a response from the authors addressing these points and clarifying the inaccuracies related to our work.

BV received honoraria and RR received royalties from Merete GmbH, Berlin Germany, producer of RigidTack. Complete disclosure of interest forms according to ICMJE are available on the article page, doi: 10.2340/17453674.2025.43005

Thank you for your attention to this matter.
